# Diagnostic Investigations as a Basis for Optimising Surgical Management of Vertebrobasilar Insufficiency Syndrome

**DOI:** 10.3389/fsurg.2022.901759

**Published:** 2022-05-16

**Authors:** Gennady Chmutin, Gennady Antonov, Egor Chmutin, Aleksei Fedyanin, Matvey Livshitz, Boris Oleynikov, Zokirzhon Zokhidov, Alina Shumadalova

**Affiliations:** ^1^ Department of Nervous Diseases and Neurosurgery, Peoples’ Friendship University of Russia (RUDN University), Moscow, Russia; ^2^ Department of Neurosurgery, 3rd Central Military Clinical Hospital named after A.A. Vishnevsky under the Ministry of Defense of the Russian Federation, Krasnogorsk, Russia; ^3^ Department of Neurosurgery, Federal State Budgetary Institution of Medical Department of Moscow Morozov Children’s City Clinical Hospital of Medical Department of Moscow, Moscow, Russia; ^4^ Department of General Chemistry, Bashkir State Medical University, Ufa, Russian Federation

**Keywords:** diagnosis of vertebrobasilar insufficiency syndrome vertebrobasilar insufficiency, diagnosis, methods, therapy, anastomosis, stroke

## Abstract

Vertebrobasilar insufficiency (VBI) is one of the most common forms of cerebrovascular pathology. The progression of the VBI, especially in the context of inadequate therapy, often leads to the formation of a persistent neurological deficits within the framework of dyscirculatory encephalopathy and the consequences of a stroke in the vertebrobasilar system. This study demonstrate the importance of objective methods of patient investigation to optimize the choice of the most effective methods of surgical treatment for VBI in cases of ineffective medical treatment. We have shown that the optimization of the diagnostic algorithm contributes to the correct individualized determination of types of surgical treatment for patients with VBI. It was found that, in addition to traditional diagnostic methods, the use of radiographic methods (ultrasound Doppler, multispiral computed tomography with contrast enhancement) is invaluable for choosing the tactics of surgical treatment. We propose a significant outcome indicator like the blood flow reactivity index to determine the postoperative improvement of blood flow in the vertebral arteries. In addition, the need to perform cerebral angiography and consultations with related specialists to exclude pathologies with a similar clinical picture is emphasized.

## Introduction

Ischemic disorders in the vertebrobasilar system occur in 30% of all cerebrovascular diseases and 70% of cases presenting with clinical features of transient ischemic attacks. Mortality from ischemic strokes in the vertebrobasilar system is twice as high as the death rate from strokes in the carotid system (1–6). Therefore, objective screening methods should help to optimize the choice of the most effective surgical treatments for vertebrobasilar insufficiency (VBI) in cases of ineffective medical treatment.

In modern studies, several prevailing factors causing narrowing of the segments of the vertebral artery (VA) and its stenosis are established, associated with age-related changes and stenosis of other major blood vessels**.** These factors make it much more difficult to identify clinical and neurological signs of vascular pathology and predetermine the use of topical diagnostics to guide treatment (7, 8). However, unified methods of localizing causes and clinical signs to determine the type of surgical treatment of VBI have not been developed. The system of diagnosing various manifestations of VA pathologies taking into account extramural compression and deformation leading to brain vascular insufficiency is not sufficiently researched (3, 5, 6, 9). We review the use of instrumental diagnostic methods for vertebrobasilar insufficiency and create an algorithm to optimize the choice of the surgical technique based on various patient characteristics.

## Materials and Methods

**Figure 1:**
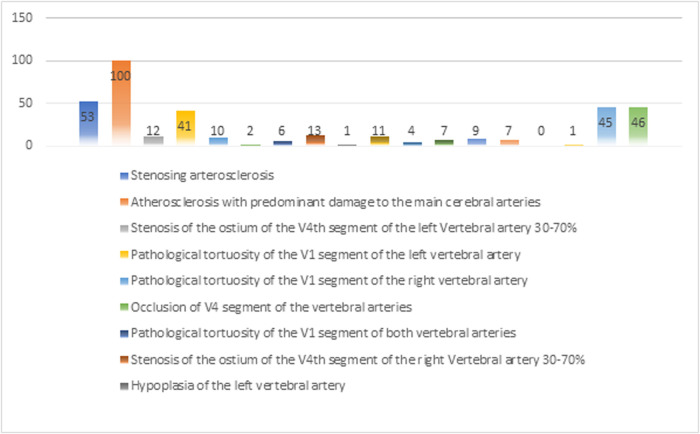
Clinical diagnosis (in % of the total).

The Study design is a single-center retrospective-prospective cohort-controlled clinical trial involving 100 patients who were treated at the 3rd Central Military Clinical Hospital named after A.A Vishnevsky (Krasnogorsk, Russia) from 2009 to 2019. In all 100 VBI patients were confirmed and various significant stenotic-occlusive lesions of the V1 segment of the VAs were identified. The presence of pathological tortuosity of the VAs was also recorded. This study was approved by the 3rd Central Military Clinical Hospital named after A.A Vishnevskyand implemented in accordance with the principles of the Helsinki Declaration (Adopted by the 18th General Assembly of the WMA). Written informed consent was obtained from all subjects. The general patient distribution by clinical diagnosis was analyzed as indicated in **Figure 1**.

As shown in **Figure 1** above, it indicates the reliability of the clinical diagnosis based on the associated clinical picture. Since the data are obtained in percentage form, it is important to note that the clinical signs of one patient could have several of these characteristics. Thus, the chances of making an error in the clinical diagnosis were eliminated. In addition, the sex-age factor was excluded, since the distribution of 100 patients by sex and age formed a comprehensive picture of the examination and the reliability of the results obtained based on the data of the sample characteristics. The average age of the patients was 72.2 years and the risk of the disease was highest between the ages of 45 and 89. In this study, 58% were men, and 42%, were women. In 50% of patients, diffuse encephalopathy syndrome (DES) was present in the vertebrobasilar system (VBS) (stage 1 DES - 1 case; stage 2 DES - 42 cases; and stage 3 DES - 7 cases), 7% had a history of transient ischemic attack (TIA) in VBS, and 4% had a history of VBI in VBS. Comorbidities were present in 98% of patients. These are presented in **Table 1**.

**Table 1:** Comorbidities identified in the study patients.

Concomitant diseases	Number of cases
Hypertension grade 1	1
Hypertensive pain grade 2	34
Hypertension grade 3	10
Diffuse encephalopathy grade 2	42
Diffuse encephalopathy grade 3	7
Diffuse encephalopathy grade 1	1
Osteochondrosis of the spine	32
Otosclerosis, mixed form	0
Parkinson syndrome	2
Ischemic heart disease, angina pectoris I	1
Ischemic heart disease, angina pectoris II	26
Ischemic heart disease, angina pectoris III	3

Depending on the leading lesion of any arterial segment involved in the blood supply to VBS in the presence of stenotic-occlusive lesions of the V1 segment of the VA, all 100 patients were divided into 2 groups: the surgical treatment group (*n* = 50) and sonservative treatment group (*n* = 50).

Choosing the type of surgical treatment for patients with VBI is an unresolved medical problem. The key difficulty in the diagnostic process lies in the multiple possible etiologies of this pathology. The process of determining the differential diagnosis is quite time-consuming and, in our opinion, this is one of the reasons for the small number of operations for patients with VBI syndrome. The problem with choosing diagnostic methods to establish the need for surgical intervention among patients with VBI is discussed by many surgeons. This is important to determine the identifying signs of this pathology, differentiating it from other conditions with a similar clinical picture (10), namely: demyelinating processes in the central nervous system, diseases of the inner and middle ear, tumors of central vestibular structures (stem, cerebellum or cortex), carotid sinus syndrome, anemia, thyroid diseases, etc. In addition, the neurosurgeon or vascular surgeon does not conduct diagnostic measures to diagnose the VBI alone, each patient requires consultation and examination by a neurologist, ophthalmologist, ENT (ear, nose, and throat) specialist, functional and radiology diagnostics specialist to exclude all “non-vascular” diseases that may mimic VBI.

**Figure 2:**
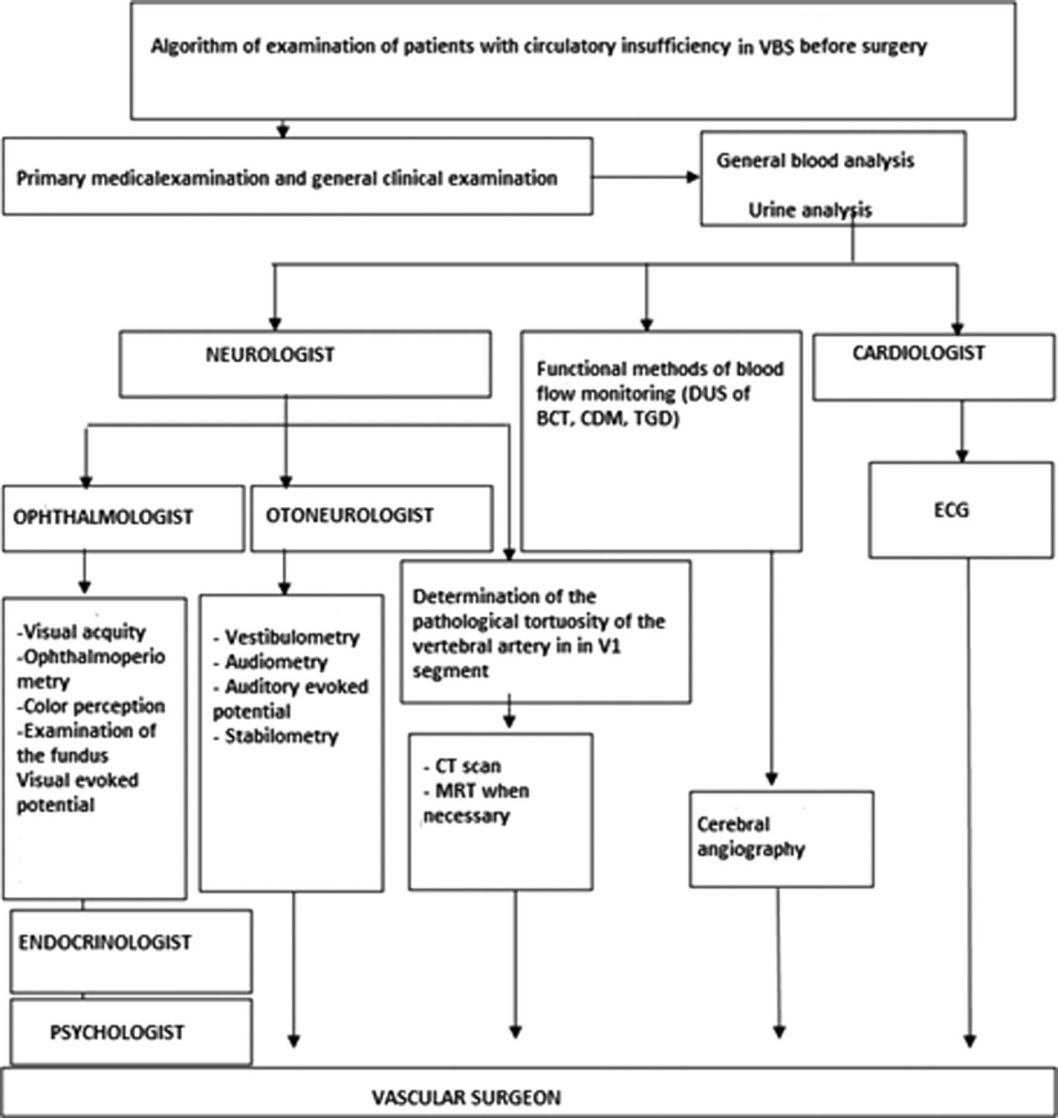
Algorithm for examination of patients with vertebrobasilar insufficiency (VBI). Note: DUS, Doppler ultrasound; BCT, Brachiocephalic trunk; VBI, Vertebrobasilar insufficiency; ECG, Electrocardiogram; CT, Computed tomography; MRT, Magnetic resonance tomography.

The primary technique of VA assessment is the study of blood flow volume to exclude VA hypoplasia. After using this algorithm Figure 2, we will analyze the procedure of patient examination by a neurosurgeon to establish the diagnosis of VBI:

**Stage 1**: General and clinical neurological examination of the patient (including the provocative test).

A. General clinical examination:
  •  Assessment of radial artery pulses or the establishment of the presence of auscultative VA murmur in the neck or supraclavicular region;
  •  Presence of blood pressure asymmetry on the hands of more than 20 mmHg.
B. Clinico-neurological examination (by a competent neurosurgeon):
  •  Compression diagnostics tests (Ethan’s test, VA compression test, dizziness test, etc.).

**Stage 2**: identifying the level of obstruction in the suspected vessels.

 - doppler u/s of the brachiocephalic artery: analysis of the diameter of arteries, velocity and volume analysis of the blood flow;
 - loading tests to determine perfusion reserve (turning and tilting of the head);
 - Analysis and calculation of the reactivity index of VA;
 - calculation of the total blood volume;
 - Transcranial doppler.

**Stage 3:** use of radiologic diagnostic methods:

 - Brain computerized tomography (CT)/magnetic resonance imaging (MRI): morphology of subtentorial and supratentorial structures of the brain;
 - CT or MRI-angiography: main arteries of the brain, anatomy of the posterior cerebral artery (PCA), excluding trifurcation of carotid arteries);
 - cerebral angiography of each segment of the VA, patency, morphological and functional status of the posterior circulation including compression tests.

## Results and Discussion

As a result of the analysis of neurological disorders, statistical indicators were determined for surgical treatment in the presence of the entire clinical complex of discoordination, vestibular-ataxia syndrome, auditory and visual disorders. Based on the proposed diagnostic algorithm, it is possible to predict the type of surgical treatment using the above indicators.

### Group 1: Anastomosis (*n* = 6)

The inclusion criteria for the anastomosis included:

1. Predominant clinical features of VBI,
2. No significant defects of the VA and subclavian artery,
3. Carotid stenosis more than 70%,
4. Lack of decompensation in the vertebrobasilar system.

The data for the clinical features of the patients in 1st group was as tabulated below (**Supplementary Figure 3****A**).

Of the 6 patients, vertebral circulation was closed (group 1.A) in 4 (66.7%) and open in 2 (33.3%) (group 1.B). Preoperative clinical manifestations of VBI in group 1 patients (*n* = 6): Dyscirculatory encephalopathy in VBI (*n* = 2, 33.3%), TIA in VBI (*n* = 1, 16.7%) and acute cerebrovascular accident in VBI (*n* = 2, 50.0%).

After full investigations, all patients underwent anastomosis surgery. **Supplementary Figure 4** indicates the cumulative survival of the patients.

### Group 2: Resection of the Tortuous Segment (*n* = 35)

Features of diffuse encephalopathy in the VB system were seen in 15 (42.9%) patients, TIA in 7 (20%) patients, 13 (37.15%) presented with acute vascular compromise in the VBS. Abnormalities of coordination, auditory and visual systems were the most common preoperatively. However, postoperatively, auditory symptoms persisted in most patients (**Supplementary Figure 3****B**).

The inclusion criteria for this group included:

1. Severe atherosclerotic lesions of the V1 segment of the VA,
2. Vertebral-subclavian steal phenomenon syndrome.

Based on the characteristics of the lesion on the V1 segment of the VA, the group was further divided into two types:

- IIA - 30 (85.7%) patients with occlusion of the V1 segment of VA,
- IIB - 15 (24.3%) patients with local, limited subtotal stenosis of the V1 segment of the VA (greater than 70%).

Both groups had reconstruction of the V1 segment of the VA. The division of patients by type of performed operations is presented in **Supplementary Figure 5**. The patients were followed up for a maximum of 3 years. The patients showed minimal improvement in the first year. However, by year 3, almost 75% of these patients had shown improvement (**Supplementary Figure 6**).

### Group 3: Reconstructive Surgery (*n* = 5)

These patients underwent Arteriolysis, X-ray endovascular stenting, and Arterioplasty.

The inclusion criteria in this group included:

1. chronic ischemia in the distribution of the VB system;
2. VA atherosclerosis in the V1 segment of the VA;
3. Bony compression of VA in 2 segments was excluded;
4. Transient ischemic attacks;
5. Stroke in the VB system.

The preoperative and postoperative clinical features in these patients are tabulated in **Supplementary Figure 3****C**.

One of the main factors for the neurosurgeon to consider when choosing the operative technique is the analysis of the V1 segment variability (11, 12), which was an exclusion factor for group 3 and an inclusion factor in group 4.

### Group 4: Resection of the tortuous segment (*n* = 4)

The inclusion criteria included:

1. See criteria for group 3;
2. coiled V1 segment of the VA;
3. High mortality risk.

Assessment of clinical features of patients in group 4 is shown in **Supplementary Figure 3****D**.

In this group, surgery on the V1 segment of the VA was performed as follows: 2 parients underwent angioplasty, stenting of the right vertebral artery (RVA), 1 patient underwent left scalenotomy, and 1 patient underwent eversion carotid endarterectomy on the left with resection of the pathological loop and redressing of the left vertebral artery (LVA).

In practice, we propose using the presented diagnostic algorithm, with the mandatory inclusion of the following elements: (1) to emphasize the need to exclude diseases with a similar clinical picture; (2) CT-angiography of the 4 segments of the VA and the basilar artery (BA); (3) analysis of the curvature of the vertebral circulation; (4) establishing the type of collateral compensation; (5) and the use of a significant outcome indicator like the blood flow reactivity index (**Supplementary Figure 7**).

The conservative treatment group which was receiving medical treatment (*n* = 50) was included to function as controls for comparison with the surgical treatment group (**Table 2**). Following the use of the above algorithm, the selected types of operations produced improvement in blood flow through the VAs in equal measure (**Table 3**).

**Table 2:** Characteristics of blood flow in the V1 vertebral artery (VA) segment depending on the type of vertebrobasilar insufficiency (VBI).

Parameters	Dyscirculatory encephalopathy	TIA in VBS	Stroke in VBS	Control group (conservative treatment)
Age at the moment of surgery	59.06 ± 8.28	69.12 ± 7.70	70.27 ± 7.60	76.12 ± 7.70
Time from the onset of the disease to surgery	4.61 ± 2.97	4.80 ± 2.84	3.97 ± 2.27	–
Blood flow to the VA, mL/min	62.13 ± 20.60	67.70 ± 22.18	63.13 ± 17.48	62.98 ± 29.11
Blood flow after the VA, mL/min	117.79 ± 23.99	116.57 ± 17.54	115. 92 ± 17.48	120.88 ± 23.54
Increase in blood flow in VA, mL/min	54.56 ± 22.33	55.96 ± 19.73	52.80 ± 17.73	55.96 ± 19.73
Total blood flow in both VAs before surgery/treatment, mL/min	207.81 ± 36.29	210.16 ± 23.37	216.16 ± 23.37	220.11 ± 28.82
Total blood flow in both VAs after surgery/treatment, mL/min	262.76 ± 36.29	264.08 ± 42.62	271.36 ± 23.99	234.18 ± 14.32
Increase in total blood flow in both Vas, mL/min	54.96 ± 30.80	53.92 ± 36.21	55.20 ± 24.96	14.07 ± 28.87
Increase in total blood flow in both VAs, mL/min	59.79 ± 23.41	61.13 ± 25.75	56.19 ± 23.09	53.65 ± 25.65
Reactivity index before surgery/treatment	0.181 ± 0.043	0.171 ± 0.070	0.197 ± 0.038	0.198 ± 0.020
Reactivity index before surgery/treatment	0.298 ± 0.068	0.296 ± 0.080	0.314 ± 0.026	0.215 ± 0.082
Increase in reactivity index	0.117 ± 0.051	0.125 ± 0.076	0.116 ± 0.042	0.017 ± 0.072

*TIA, Transient ischemic attack; VBS, Vertebrobasilar system; –, Not determined.*

**Table 3:** Characteristics of blood flow in the V1 segment of vertebral artery (VA). depending on the type of surgery performed.

Parameter	Transition of the VA to CCA (*n* = 67)	Anastomosis	Resection of tortuosity	Reconstructive surgery
	M ± SD	M ± SD	M ± SD	M ± SD
Age at the moment of surgery	60.45 ± 8.66	69.97 ± 7.33	76.46 ± 8.05	70.09 ± 6.94
Time from the onset of the disease to surgery	4.73 ± 3.28	4. 53 ± 2.73	4.50 ± 1.80	3.69 ± 1.99
Blood flow to the VA, mL/min	65.08 ± 18.34	58.12 ± 19.10	75.11 ± 21.52	57.48 ± 15.83
Blood flow after the VA, mL/min	120.83 ± 20.60	111.52 ± 18.38	118.71 ± 24.63	113.95 ± 17.21
Increase in blood flow in VA, mL/min	55.18 ± 23.42	52.14 ± 16.38	46.29 ± 18.19	56.48 ± 17.30
Total blood flow in both VAs before surgery/ treatment, mL/min	214.27 ± 22.59	209.35 ± 25.63	212.96 ± 26.24	211.49 ± 24.24
Total blood flow in both VAs after surgery/treatment, mL/min	271.33 ± 26.23	252. 74 ± 252.74	271.14 ± 30.37	268.91 ± 20.25
Increase in total blood flow in both Vas, mLl/min	59.26 ± 23.20	57. 00 ± 21.46	58.18 ± 27.29	57.42 ± 23.43

*CCA, Common carotid artery; M, Mean; SD, Standard deviation.*

When analyzing the volumetric blood flow rates, it was noted that among the operated patients, a decrease in the clinical features of VBI was observed in patients with an increase in the total volumetric velocity of blood flow in the VA of more than 250 mL/min (*p* < 0.05). An increase in the total volumetric blood flow of more than 250 mL/min was observed in most patients after various types of operations on the 1st segment of the VA.

Favorable outcomes including a decrease or disappearance of the clinical features of VBI were observed in most patients with an increase in total volumetric blood flow through the VA to 250 mL/min or more, and unfavorable outcomes i.e. deterioration or return of the clinical features of VBI was seen in patients with post-surgical total volume blood flow in the VA of less than 250 mL/min and all patients of the control group.

## Conclusion

The use of the diagnostic algorithm contributes to the accurate prediction of the type of surgical intervention for various patients with vertebrobasilar insufficiency. In addition to traditional diagnostic methods, the gold standard investigations to guide the surgical intervention are radiological investigations including ultrasonic Doppler, contrast-enhanced multispiral CT, and CT-angiography. The need to perform cerebral angiography and multidisciplinary specialist consultations to exclude pathologies with a similar clinical picture cannot be overemphasized.

## Data Availability

The original contributions presented in the study are included in the article/**Supplementary Material**, further inquiries can be directed to the corresponding author/s.
