# Supplementary figures and images for: Diagnostic Investigations as a Basis for Optimising Surgical Management of Vertebrobasilar Insufficiency Syndrome
Source: Front Surg. 2022 May 16;9:901759. doi: 10.3389/fsurg.2022.901759 (PMC9150087; doi:10.3389/fsurg.2022.901759)

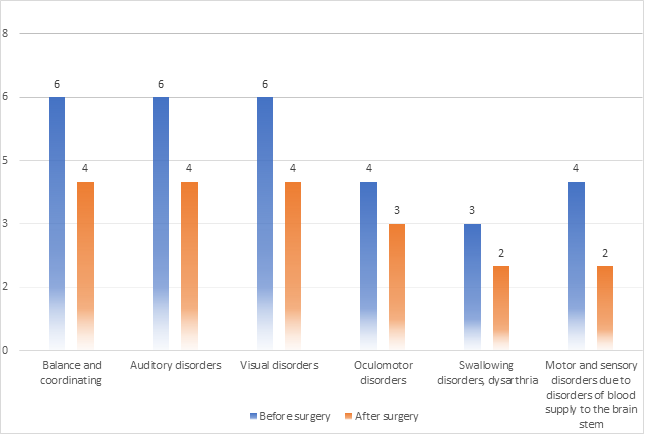

Supplement: Supplementary file 1 [file Data_Sheet_1_v2.zip › 901759 supplementary/Supplementary Material/FIG 1A Supp.tif]

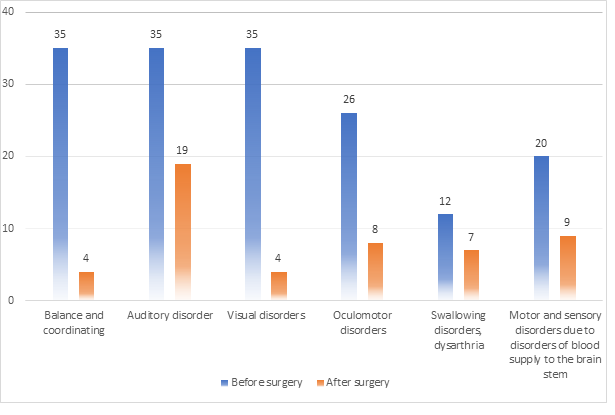

Supplement: Supplementary file 1 [file Data_Sheet_1_v2.zip › 901759 supplementary/Supplementary Material/FIG 1B Supp.tif]

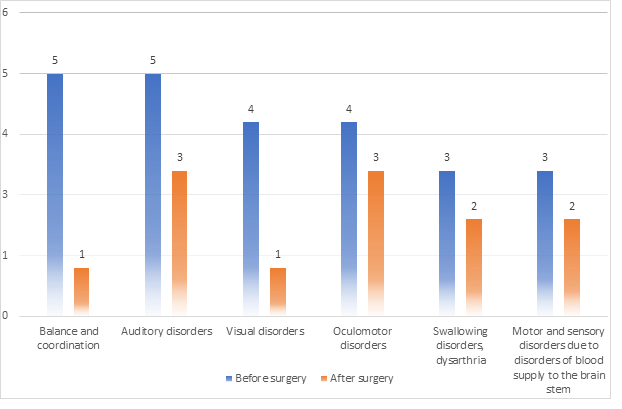

Supplement: Supplementary file 1 [file Data_Sheet_1_v2.zip › 901759 supplementary/Supplementary Material/FIG 1C Supp.tif]

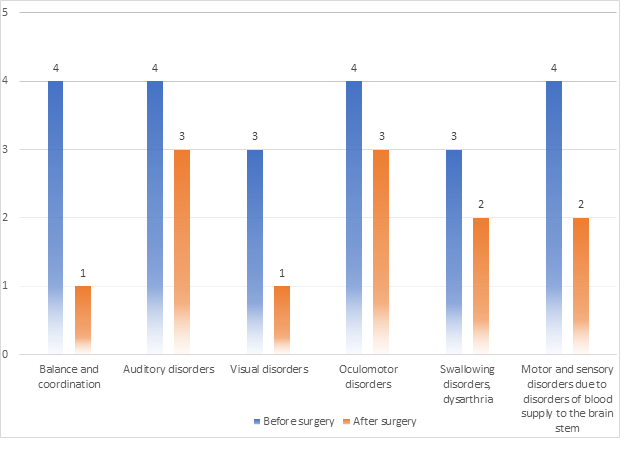

Supplement: Supplementary file 1 [file Data_Sheet_1_v2.zip › 901759 supplementary/Supplementary Material/FIG 1D Supp.tif]

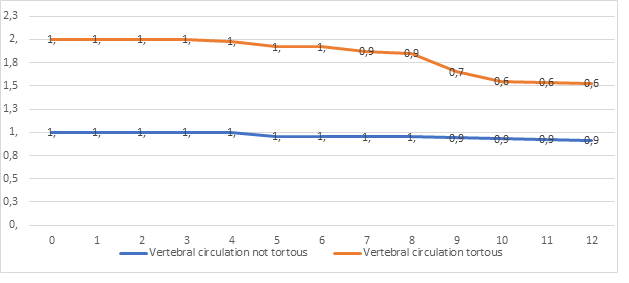

Supplement: Supplementary file 1 [file Data_Sheet_1_v2.zip › 901759 supplementary/Supplementary Material/FIG 2 Sup.tif]

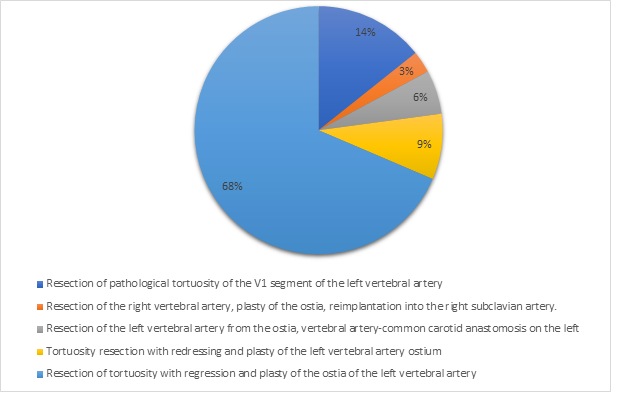

Supplement: Supplementary file 1 [file Data_Sheet_1_v2.zip › 901759 supplementary/Supplementary Material/FIG 3 Sup.tif]

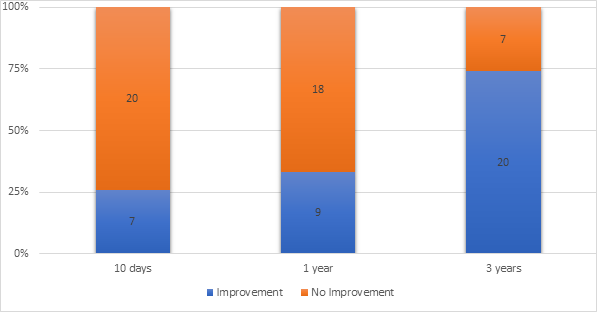

Supplement: Supplementary file 1 [file Data_Sheet_1_v2.zip › 901759 supplementary/Supplementary Material/FIG 4 Sup.tif]

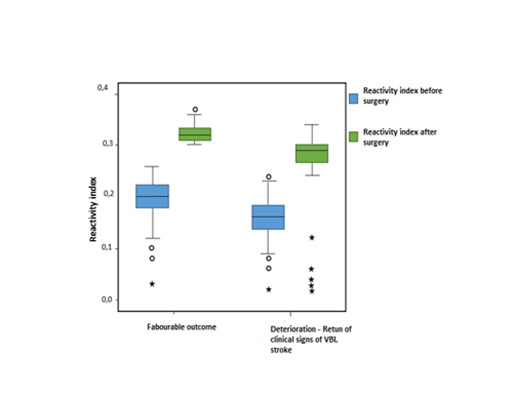

Supplement: Supplementary file 1 [file Data_Sheet_1_v2.zip › 901759 supplementary/Supplementary Material/FIG 5 Sup.tif]
